# Cystic Fibrosis and Beckwith-Wiedemann Syndrome: A Case Report

**DOI:** 10.14740/jocmr2003w

**Published:** 2014-12-29

**Authors:** Claudia Aguiar, Liane Correia-Costa, Paulo Eden, Luisa Guedes-Vaz

**Affiliations:** a Pediatric Department, Hospital Pediatrico Integrado, Centro Hospitalar Sao Joao, Porto, Portugal; b Division of Pediatric Pneumology, Hospital Pediatrico Integrado, Centro Hospitalar Sao Joao, Porto, Portugal

**Keywords:** Cystic fibrosis, Beckwith-Wiedemann syndrome, Genetic diseases

## Abstract

Cystic fibrosis (CF) is a hereditary disease of exocrine gland function that involves multiple systems but chiefly results in chronic respiratory infections, the major cause of death, pancreatic enzyme deficiency and severe malnutrition, mostly in untreated patients. The association between CF and other inherited diseases or congenital anomalies is rare. We describe for the first time the association of CF and Beckwith-Wiedemann syndrome (BWS). BWS is a genetic disorder commonly characterized by overgrowth. The most common features of BWS include macrosomia, macroglossia, abdominal wall defects, an increased risk for childhood tumors, kidney abnormalities, hypoglycemia in the newborn period and unusual ear creases or pits.

## Introduction

Cystic fibrosis (CF; OMIM 219700) is an autosomal recessive disorder caused by mutations in the cystic fibrosis transmembrane conductance regulator (CFTR) gene, which encodes a protein expressed in the apical membrane of exocrine epithelial cells [1-4]. These mutations result in dysfunction of the apical membrane CFTR protein, affecting the chloride and sodium transport in secretory epithelial cells with abnormal ion concentrations across the apical membranes of these cells. CFTR dysfunction results in ionic imbalance of epithelial secretions in several organs, such as pancreas, gastrointestinal tract, liver, reproductive and respiratory systems. So the clinical manifestations include progressive pulmonary damage leading to respiratory failure, pancreatic dysfunction, liver disease that may progress to cirrhosis, gut motility problems, male infertility and high sweat electrolytes [1-3, 5, 6].

Simultaneous occurrence of CF and other inherited diseases or congenital anomalies has been seldom described [7]. We report a case of a 11-year-old girl with the diagnosis of CF and Beckwith-Wiedemann syndrome (BWS; OMIM 130650). This is a rare overgrowth disease that is characterized by prenatal and postnatal macrosomia, macroglossia and abdominal wall defects. Other signs are neonatal hypoglycemia, hemihyperplasia, ear anomalies, cleft palate, polydactyly, visceromegaly, renal abnormalities, adrenocortical cytomegaly, an increased risk of embryonic tumors and a positive family history [8-10]. BWS is associated with abnormalities of chromosome 11p15, including epigenetic changes, paternal disomy and point mutations [10-13].

## Case Report

The patient is her parents’ only child. They are young, Caucasian and not family related. She was born after 37 weeks of gestation, delivery by cesarean section because of macrosomia. Antenatal ultrasound at the third trimester revealed macrosomia, omphalocele, macroglossia, hepatosplenomegaly and fetal and placentar calcifications. Birth weight was 4,540 g (> 97th percentile), height of 53 cm (97th percentile) and head circumference 34.5 cm (50th - 85th percentile). Apgar score was 9 and 10 at 1 and 5 min, respectively. At birth she presented classical BWS features.

A neonatal abdominal computed tomography (CT) scan showed also a congential malformation of pielocalicial system, with probable tubular ectasia, and renal micronodules. The correction of the omphalocele was performed at the fourth day of life. When she was 3 months old, a partial resection of the tongue was performed and at 2 years of life, she had a transcatheter intervention for treatment of patent ductus arteriosus. She had several respiratory infections with wheezing, and when she was 9 years old, she underwent lung function that showed a mixed pattern, restrictive and obstructive. She also had a thoracic CT scan with fibrotic grooves and bronchiectasis in the right upper lobe, middle lobe, lingula and superior segment of the right lower lobe. At this level some micronodules suggestive of infection with endobronchial dissemination or mucoid impaction were visualized. A sweat test was performed and was positive (107 mmol/L). CF genotyping results were positive for G542X/G85E mutations. In addition, the patient was found to have a severely low fecal elastase level consistent with pancreatic insufficiency.

Meanwhile, the patient who is now 11 years old, has required eight hospitalizations to manage complications of CF, namely two pancreatitis and lung infections.

Methylation analysis of the two domains at 11p15.5, DMR1 (H19) and DMR2 (KCNQ1OT1) was performed by methylation-specific MLPA. Hypomethylation at DMR2 (KCNQ1OT1) and hypermetilation of DMR1 (H19) were identified. This is signal of uniparental paternal dissomy. Patogenic alterations in the analized region of CDKN1C gene were not found.

## Discussion

Few reports have described the association of CF with other inherited or congenital diseases. These reports include association with congenital generalized hamartoma [14], infantile hypertrophic pyloric stenosis [15], intra sylvian fibroma [16], gastro-intestinal cancer [17], syringomyelia and scoliosis [18], lymphoblastic leukemia [19], sickle cell disease [7, 20-22], congenital adrenal hyperplasia [7], and Ehler’s Danlos syndrome [7, 23].

We describe for the first time the association of CF and BWS.

BWS is etiologically heterogeneous arising from dysregulation of one or both of the chromosome 11p15.5 imprinting centers and/or imprinted growth regulatory genes on chromosome 11p15.5. Most BWS cases are sporadic and result from loss of maternal methylation at imprinting center 2 (DMR2), gain of maternal methylation at imprinting center 1 (DMR1) or paternal uniparental disomy [11]. In this case we found an 11p15.5 paternal uniparental disomy which corresponds to 20% of patients with BWS [24].

In Portugal, we have one CF patient in 6,000 neonates. BWS is even less frequent. The association between these two diseases is an interesting and never yet described situation. Because of the risks and everyday problems related to two severe diseases in the same patient (infections, malnutrition, possibility of malignant tumors, etc.), her management is and will be a never ending challenge.
